# Cognitive control in the self-regulation of physical activity and sedentary behavior

**DOI:** 10.3389/fnhum.2014.00747

**Published:** 2014-09-29

**Authors:** Jude Buckley, Jason D. Cohen, Arthur F. Kramer, Edward McAuley, Sean P. Mullen

**Affiliations:** ^1^School of Psychology, University of AucklandAuckland, New Zealand; ^2^Department of Kinesiology and Community Health, University of Illinois at Urbana-ChampaignUrbana, IL, USA; ^3^Beckman Institute for Advanced Science and TechnologyUrbana, IL, USA

**Keywords:** cognitive control, self-regulation, executive functioning, physical activity, sedentary behavior

## Abstract

Cognitive control of physical activity and sedentary behavior is receiving increased attention in the neuroscientific and behavioral medicine literature as a means of better understanding and improving the self-regulation of physical activity. Enhancing individuals’ cognitive control capacities may provide a means to increase physical activity and reduce sedentary behavior. First, this paper reviews emerging evidence of the antecedence of cognitive control abilities in successful self-regulation of physical activity, and in precipitating self-regulation failure that predisposes to sedentary behavior. We then highlight the brain networks that may underpin the cognitive control and self-regulation of physical activity, including the default mode network, prefrontal cortical networks and brain regions and pathways associated with reward. We then discuss research on cognitive training interventions that document improved cognitive control and that suggest promise of influencing physical activity regulation. Key cognitive training components likely to be the most effective at improving self-regulation are also highlighted. The review concludes with suggestions for future research.

For nearly half of a century, researchers have been trying to uncover how to motivate people to become more physically active (Trost et al., 2002; Schutzer and Graves, 2004; Buckworth et al., 2013) and, recently, more effort has been made to understand how to motivate people to be less sedentary (Hamilton et al., 2008). Despite resources devoted to these efforts, more than 30% of the world’s population remains physically inactive (Hallal et al., 2012) and, on average, people are sitting for more than 300 min/day (Bauman et al., 2011). Our understanding of the regulation of these behaviors has advanced, but these prevalence rates suggest that our knowledge of physical activity and sedentary behavior remains incomplete. Research supports theoretical proposals that health behavior is dependent, in part, on self-regulation capacities (Bandura, 1986; De Ridder and de Wit, 2006), but only recently has research attention been directed toward the preceding factors of self-regulation that influence physical activity and sedentary behavior.

Recent theory (e.g., Temporal Self-Regulation Theory; Hall and Fong, 2007, 2010, 2013) and evidence suggest that the relation between physical activity and cognitive control is reciprocal (Daly et al., 2013). Most research has focused on the beneficial effects of regular physical activity on executive functions-the set of neural processes that define cognitive control. Considerable evidence shows that regular physical activity is associated with enhanced cognitive functions, including attention, processing speed, task switching, inhibition of prepotent responses and declarative memory (for reviews see Colcombe and Kramer, 2003; Smith et al., 2010; Guiney and Machado, 2013; McAuley et al., 2013). Recent research demonstrates a dose-response relationship between fitness and spatial memory (Erickson et al., 2011), however the long-term effects of physical activity on working memory have been less consistent (Smith et al., 2010).

Positive physical activity effects on executive function have been found in children for both acute and regular activity (Chang et al., 2012; Hillman et al., in press). For example, findings from a 9-month randomized controlled trial in 221 prepubertal children attending an afterschool physical activity program (vs. a wait-list control group), showed improvements in fitness (VO_2peak_), cognitive control, and neuroelectrical activity (P3-ERP) during tasks that required significantly more cognitive control (Hillman et al., in press). In addition, a modest dose-response effect of program attendance on cognitive control measures was also found. Improvements in cognitive function are not always observed in older adults (Angevaren et al., 2008) or in children (Janssen et al., 2014) involved in physical activity programs. These findings suggest that the effects of physical activity on cognitive function may depend on the particular cognitive function being assessed. Taken together, this research suggests that physical activity training can enhance cognitive control abilities. The effects of physical activity on cognitive control appear to be underpinned by a variety of brain processes including: increased hippocampal volume, increased gray matter density in the prefrontal cortex (PFC), upregulation of neurotrophins and greater microvascular density (for a review see Voss et al., 2013). Much less is understood about the influence of cognitive control on physical activity but emerging evidence suggests that executive functions play an antecedent role in effective self-regulation of physical activity (Hall et al., 2008; Riggs et al., 2010; McAuley et al., 2011; Daly et al., 2013; Pentz and Riggs, 2013; Best et al., 2014).

The goals of this paper are (1) to review emerging evidence of the antecedence of cognitive control abilities in enabling successful self-regulation for physical activity, and in precipitating self-regulation failures that predispose individuals to remain sedentary; (2) to highlight neural networks that may underlie the cognitive control of physical activity and sedentary behavior; and (3) to review emerging research on training effects on cognitive and physical functioning and summarize components of training that may produce positive cognitive outcomes associated with increased physical activity engagement.

## Self-regulation, physical activity and sedentary behavior

Health behavior is determined from the interplay between personal influences, behavior and environmental factors (Bandura, 2004). Although external circumstances play an important role in facilitating or impeding physical activity (for reviews see French et al., 2001; Trost et al., 2002) and sedentary behavior (Owen et al., 2010), to a large degree, external factors influence behavior through the mediation of a person’s cognitive processes (Bandura, 2001). Executive functions, in particular, are pivotal to cognitive control. Executive function is an umbrella term encapsulating a variety of higher-order cognitive processes that regulate, control, and modulate information from many cortical-subcortical brain regions to support goal-directed behavior (Blair and Ursache, 2011; Otero and Barker, 2014). Executive functions and the capacity to symbolize enable individuals to self-regulate by internal representations regarding future goal-directed actions (Bandura, 2001; Barkley, 2001). From this perspective, internal representations of future consequences allow people to exert adaptive control anticipatorily over their behavior and thereby manipulate, alter, and influence their environments. Rapid technological and societal evolution has reduced the function of physical activity as a requisite and inherent part of everyday life (Conroy et al., 2010). These changes have increased the importance of cognitive control capacities for the self-regulation of physical activity for health in contemporary society. Self-regulatory systems operate through goals, beliefs, self-monitoring, evaluative self-reactions, and self-regulatory processes (Bandura, 1986, 1997; Suchy, 2009; Hagger, 2010; Hall and Fong, 2010). Self-regulation implies the modulation of thought, affect, behavior, or attention via deliberate or automated use of cognitive control mechanisms (Karoly, 1993). Given the intrinsic plasticity of the cognitive control system (Anguera et al., 2013) it is possible that advanced cognitive control capacities are underpinned by brain networks specialized for self-regulation.

Short- and long-term self-regulation success can be enhanced with control over limited executive attentional resources (Sethi et al., 2000; Rueda et al., 2005). Attention biases seem to differ as a function of physical activity (Hawkins et al., 1992; Kramer et al., 1999a). For instance, physically active people have stronger cognitive and evaluative biases toward physical activity-related concepts than less physically active individuals (Calitri et al., 2009; Conroy et al., 2010; Hyde et al., 2010; Berry et al., 2011). Strong evaluative biases have been prospectively associated with objectively measured physical activity behavior (Conroy et al., 2010). Also, experienced exercise self-regulators display non-conscious biases towards exercise self-regulatory processes (Buckley and Cameron, 2011a), and towards associating exercise self-regulatory concepts (e.g., self-efficacy) as self-relevant compared to those with less experience (Buckley and Cameron, 2011b). In contrast, less experienced exercise self-regulators appear biased towards associating exercise disengagement as self-relevant, and sedentary individuals, display negative biases toward physical activity (Bluemke et al., 2010). Together, this research suggests that an improvement in control processes, such as attention and inhibition or interference control, is associated with an improvement in self-regulation of physical activity.

## Cognitive control and self-regulation of physical activity

Self-regulation capacity is intricately linked to executive functions (Hofmann et al., 2012). Core executive functions include updating and monitoring of relevant information in working memory, inhibitory control, including self-control (behavioral inhibition) and interference control (selective attention and cognitive inhibition), planning, scheduling, and flexible switching between different tasks or mental sets (set-shifting; cognitive flexibility; for reviews see Miyake et al., 2000; Lehto et al., 2003; Diamond, 2013). These functions afford the cognitive control that enable individuals to maintain goals across prolonged periods of time, flexibly adapt behavior to changing demands and detect conflict and discrepancy and adjust behavioral control accordingly (Botvinick et al., 2001; Braver and Barch, 2006). Cognitive control abilities, therefore, partly underlie the capacity for self-regulation.

People with greater self-regulation capacity engage in more healthful behaviors and are more successful at implementing their intentions to be physically active (de Bruin et al., 2012). Efficient executive functions support self-regulatory mechanisms that underpin successful goal pursuit (Hofmann et al., 2012). For instance, people with high working memory capacity are more adept at sustaining attention on a focal task (e.g., Engle, 2002) and are better able to resist attentional capture by distracting stimuli at early stages of self-regulation processing (Friese et al., 2010), have greater ability to inhibit intrusive thoughts (Brewin and Beaton, 2002) and are more successful at downregulating unwanted “hot” processes such as, negative affect and cravings (Gyurak et al., 2012). Moreover, people that are effective at inhibiting habitual, prepotent responses are more successful at suppressing, ignoring or disengaging from distracting information that might interfere with their self-regulatory efforts (Hofmann et al., 2008).

Increasingly, the primacy of cognitive control abilities in physical activity behavior is receiving strong empirical support (e.g., Hall et al., 2006; Riggs et al., 2010; McAuley et al., 2011; Daly et al., 2013; Pentz and Riggs, 2013). Of particular note, Hall et al. (2008) demonstrated that individual differences in executive function uniquely predict physical activity behavior. Specifically, greater baseline levels of inhibition control, as indicated by better performance on a reaction time measure of executive function (Go/NoGo task) was associated with physical activity over a subsequent 7-day period. McAuley et al. (2011) examined the relationship of executive function, self-regulation and self-efficacy in adherence to a 12-month exercise intervention for older adults. McAuley et al. found that at the start of the exercise program, higher levels of executive ability to coordinate tasks and inhibit habitual responses together with greater use of self-regulatory strategies were associated with higher levels of exercise self-efficacy. In turn, higher self-efficacy was linked to better adherence to weekly physical activity during the ensuing 11 months. Together, this research suggests that at the start of an exercise program, stronger cognitive control abilities are directly and indirectly associated with increased physical activity behavior. Furthermore, in a multi-wave longitudinal study investigating the reciprocal relationship between executive function and physical activity in 4555 older adults (Daly et al., 2013), it was found that changes in executive function corresponded with changes in physical activity. High levels of executive function predicted a longitudinal increase in physical activity. However, older adults with poor executive function tended to show large decreases in their rates of participation in physical activity over time. The magnitude of the relationship between physical activity and cognitive performance was shown to be strongest in the direction from executive function to physical activity. These findings suggest that with increasing age, executive function plays a predictive role in physical activity participation. Similarly, findings from research examining the relationship between executive function and physical activity in children show that poor performance on executive function tasks prospectively predict low levels of physical activity (Riggs et al., 2010; Pentz and Riggs, 2013).

## Cognitive control and sedentary behavior

When self-regulation capacities are limited, i.e., fatigued, underdeveloped or disengaged, health behaviors are more strongly influenced by subtle, transient behavioral prepotency effects and temptations (Hall and Fong, 2007; Pontus Leander et al., 2009). Without efficient cognitive control abilities, people are more likely to give in to temptations when exposed to them and are predisposed to undermine their self-regulatory functioning. Limited self-regulatory capacity is associated with overeating, smoking, drug use, unsafe sex and low adherence to physical activity (Ayduk et al., 2000; Tarter et al., 2003; Bogg and Roberts, 2004; Nigg et al., 2006; Hagger, 2010). Relatively lower cognitive control increases the likelihood that individual (e.g., bad mood) and situational (e.g., cognitive load) risk factors will contribute to self-regulatory failures (Hofmann et al., 2012). For example, individuals with low working memory capacity have greater difficulty overcoming attentional biases (Friese et al., 2010) and are less able to resist attending to distractors (Unsworth et al., 2004), are more vulnerable to intrusive thoughts (Brewin and Smart, 2005) and unintended mind wandering (Kane et al., 2007). People with relatively lower cognitive control are also less able to disengage from affective reactions towards temptations (Hofmann et al., 2008) and find it difficult to inhibit or override impulsive behavior responses (Hofmann et al., 2009).

In daily life, people often pursue physical activity goals in the face of multiple alternative goals (e.g., work goals) and temptations that compete for limited cognitive resources. These goal conflicts and temptations can impose a self-regulation dilemma (Fishbach and Zhang, 2008). Recent evidence suggests that executive functions may play a causal role in resolving competing health behavior influences (Hall and Marteau, 2014). Cognitive control abilities, such as effective working memory operations, allow people to proactively maintain accessibility of physical activity goals and reactively resolve competition by inhibiting competing interests, thereby reducing the risk that they will give in to sedentary temptations and predispositions.

Sedentary behavior is a distinct construct from physical activity and has independent and qualitatively different effects on health and physical function (Hamilton et al., 2008; Katzmarzyk et al., 2009; Tremblay et al., 2010). Sedentary behavior is defined as any waking behavior characterized by a low energy expenditure of less than or equal to 1.5 metabolic equivalents (METs) while in a sitting or reclining posture (Tremblay et al., 2012; Sedentary Behavior Research Network, 2014). Sedentary behavior can be categorized into transformational (e.g., driving), occupational (e.g., using a computer), and/or leisure (e.g., watching TV; Voss et al., 2014). Research suggests that chronic, unbroken periods of muscular unloading associated with prolonged sedentary time is linked with increased all-cause mortality and risk of chronic diseases (Hamilton et al., 2007, 2008), and with deleterious health outcomes associated with cognition and brain health (Voss et al., 2014). Prolonged sitting has been shown to be associated with increased premature death risk after controling for factors such as age, smoking status, gender, education, body mass index and living in urban or city environments (van der Ploeg et al., 2012). Interruptions to sitting time have been shown to have metabolic-health benefits (Dunstan et al., 2011). Much less is understood about the regulatory determinants of sedentary behavior, and very few studies have explicitly examined the relationship between executive function and sedentary behavior. Nonetheless, there is preliminary evidence that lower executive function is directly and indirectly associated with sedentary behavior. For example, Hoang et al. (2013) found that young adults who initially exhibited low levels of physical activity and remained relatively inactive for 25 years had nearly twofold greater odds of impaired executive function compared with those who exhibited higher activity levels; very-low physical activity patterns were associated with even more pronounced declines in executive functioning. Similarly, in older adults, sedentary behavior indirectly led to poor executive function through depressive symptoms (Vance et al., 2005). Sedentary individuals have also been shown to have inefficient task switching abilities as indicated by greater switch costs compared to those that are more active (Hawkes et al., 2013). That is, sedentary individuals display less capacity to quickly and accurately switch between tasks. In a study of 9–10 year-old children, executive control proficiency was negatively associated with sedentary behavior possibly reflecting a lack of cognitive capacity to plan exercise and/or to regulate urges to remain sedentary (Riggs et al., 2012).

In addition to keeping goals in mind and inhibiting habitual, unhealthy responses, effective self-regulation requires cognitive flexibility, i.e., the ability to shift between multiple tasks or mental sets (Monsell, 2003). Prior research has established that in switching from one task to another, the two executive control processes of goal shifting and rule activation each take time (Rubinstein et al., 2001). Smaller switching costs reflect more efficient executive functioning (Monsell, 2003). Although the connection between task switching and self-regulation has received less attention, findings from a randomized controlled trial in older adults, demonstrated that task switching abilities improved with concurrent increases in cardiorespiratory fitness (Kramer et al., 1999b). The extent to which task-switching promotes or impedes self-regulation is a function of task context and motivational factors (Hofmann et al., 2012). Given that mental set—an individuals’ tendency to repeatedly approach a situation in the same way—is a precursor to habit (Galarce and Kawachi, 2013), the ability to change perspectives or to rapidly adjust strategies in line with changing demands or priorities is likely a prerequisite for engaging in regular physical activity. Together, the discussed research suggests that executive control abilities affect the emergence as well as the regulation of physical activity and sedentary behavior. Furthermore, in line with recent neuroscience models (e.g., Power et al., 2011), self-regulation of physical activity and sedentary behavior is likely implemented via dynamic and flexible “control” neural networks.

## Neural networks of cognitive control and self-regulation

Recent advances in neuroscience research demonstrate that cognitive control emerges from multiple, distinct, functional brain networks that flexibly interact in the service of goal directed action (Bressler, 1995; Goldman-Rakic, 1995; McIntosh, 2000; Mesulam, 2005; Fuster, 2006; Fair et al., 2009; Bressler and Menon, 2010). Within broadly distributed networks, dynamic functional connectivity between brain regions specialized to support self-reflection, cognitive control and those associated with the salience, reward and emotional value of a stimulus provide the neural underpinnings of goal-directed behavior (Fuster, 2001; Pochon et al., 2002; Aron et al., 2004; Banfield et al., 2004; Vincent et al., 2008; Menon and Uddin, 2010; Spreng et al., 2010; Heatherton and Wagner, 2011).

Making a decision to engage in physically active or sedentary behaviors likely activates a variety of brain regions that continuously compete to determine the direction of a person’s thoughts and behavior. Indeed, evidence suggests that competing brain regions are activated in decisions involving the physical and mental effort cost associated with an action (Botvinick et al., 2009). Activity in prefrontal regions associated with cognitive control (e.g., the dorsal anterior cingulate cortex (dACC)) and subcortical regions associated with reward (e.g., amygdala, ventral striatum, mesolimbic dopamine pathway) have been shown to vary when people make decisions about whether an action is worth taking relative to the physical and mental effort they are required to invest (Walton et al., 2003, 2006; Kurniawan et al., 2010). Cognitive control mechanisms are crucial for “biasing” neural competition in favor of goal-directedness (Miller and Cohen, 2001; Blair and Ursache, 2011; Hanif et al., 2012). By amplifying activity in neural structures responsible for processing goal-relevant information and attenuating/suppressing competing neural activity, executive functions direct attention selectively towards self-regulatory cues, making them more likely to guide behavior.

To our knowledge, no research has focused on the brain networks responsible for the self-regulation of physical activity. Nonetheless, choosing to engage in physical activity requires the capacities to engage internally focused thoughts about physical activity as well as make decisions based on long term rewards, disengage from cues associated with more immediate outcomes and override long standing, but interfering habits. Thus, the self-regulation of physical activity likely shares conceptual similarities with other domains of intertemporal choice that depend upon the integration of cognitive control abilities (e.g., healthy eating, smoking cessation). Cognitive control mechanisms have been increasingly implicated in the self-regulation of a wide range of health behaviors (Insel et al., 2006; Nes et al., 2009; Williams et al., 2009; Hall, 2012; Jasinska et al., 2012), and emerging literature shows that baseline levels of these abilities improve with training (Olesen et al., 2004; Erickson et al., 2007; Dahlin et al., 2008). This research implies that multiple types of self-regulation may engage overlapping neural networks (Aron et al., 2004; Cohen and Lieberman, 2010; Cohen et al., 2013). Evidence that PFC regions are consistently recruited in exerting control across different self-regulation domains, but subcortical regions of this top down control are dependent on the nature of the stimulus and the regulatory context offers support for this idea (Cohen and Lieberman, 2010; Heatherton and Wagner, 2011). Self-regulation therefore, requires a balance between prefrontal and subcortical brain networks.

**Figure 1:**
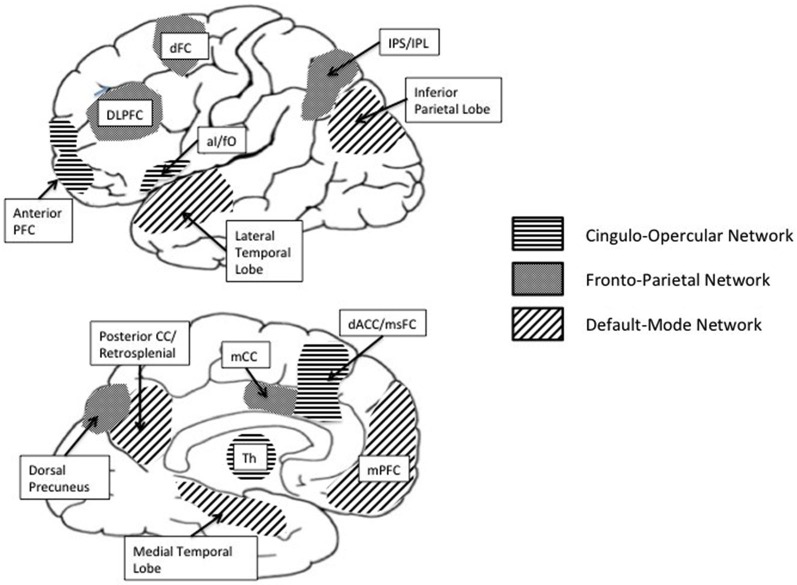
**Brain networks associated with cognitive control**.

Building on these neuroscience developments, it is plausible that the self-regulation of physical activity and sedentary behavior is implemented via overlapping networks of brain regions (see Figure 1) including (1) the default mode network (DMN) involved in resting states and internal thought (Andrews-Hanna, 2012); (2) PFC regions involved in cognitive control—particularly, the “fronto-parietal” network implicated in initiation and flexible adjustments in control and the “cingulo-opercular” network implicated in prolonged maintenance of task context (Dosenbach et al., 2007, 2008); and (3) brain regions and pathways associated with reward (e.g., ventromedial prefrontal cortex (vmPFC), amygdala, ventral and dorsal striatum, mesocortical and mesolimbic) (McClure et al., 2004; Draganski et al., 2008; Van Leijenhorst et al., 2010).

## Self-reflection and physical activity

In contemplating physical activity, people often engage in introspection on their aspirations for exercise, planning exercise sessions, and evaluating exercise goals and discrepancies. During these periods of self-reflection, the DMN, a set of interconnected brain regions including the medial prefrontal cortex (mPFC) and posterior cingulate cortex (PCC), the retrosplenial cortex (RSC), the medial and lateral temporal lobes, and the posterior inferior parietal lobes (pIPL) is engaged (Northoff et al., 2006; Buckner et al., 2008; Stawarczyk et al., 2011; Moran et al., 2013). Although activity in the DMN has been most often associated with resting states, recent research has implicated activity in the DMN with simulations of internal experience including mind wandering and the recollection of personal preferences, beliefs, feelings and abilities (Ochsner et al., 2004; Amodio and Frith, 2006; van Overwalle, 2009; Andrews-Hanna, 2012). Mind wandering can promote or impede goal directed behavior (Smallwood and Andrews-Hanna, 2013). For instance, mind wandering is associated with adaptive self-regulatory skills such as, planning, problem solving and delay of gratification (Schacter et al., 2007; Suddendorf and Corballis, 2007; Suddendorf et al., 2009), and with a style of decision-making characterized by patience rather than impulsivity (Smallwood et al., 2013). These regulatory processes enable people to select optimal courses of action to achieve their goals. Mind wandering can also manifest as self-regulation failures in situations where people experience goal neglect. In such situations, unintended lapses of attention have been shown to lead people to automatically engage well-established habits instead of acting in accordance with current goals (McVay and Kane, 2009, 2010). Given the association of mind wandering with self-regulatory success and failure, it seems plausible that in physically active individuals, mind wandering may be linked to thoughts about planning or overcoming physical activity barriers that aid self-regulatory efforts whereas, in individuals that remain sedentary, mind wandering may trigger thoughts that lead them to neglect their physical activity goals.

Research on the relationship between the DMN and executive function demonstrates that increased activity in the DMN is associated with increased memory capacity in young adults (Hampson et al., 2006) and improved performance on a variety of tasks that assess cognitive control in older adults (Persson et al., 2007; Voss et al., 2010). Voss et al. reported that increased functional connectivity within the DMN, and between the DMN and a frontal executive network, is associated with aerobic fitness and with activities that appear to engage executive functions required for learning exercise routines. The DMN shows reduced activity during cognitive tasks that require externally focused goal-directed attention (Fox et al., 2005; Fox and Raichle, 2007).

## Translating mentally represented goals to behavior

Translating internally represented physical activity-related goals to action requires people to keep goals in mind, direct attention away from distractors, inhibit competing impulses and make decisions about how to proceed. These cognitive processes are subcomponents of executive functions and are associated with increased activity in prefrontal brain networks that exert a supervisory function that governs the regulation of behavior (Bickel et al., 2012). Regulation of physical activity behavior likely requires cognitive control optimized for both rapid adaptive control via the fronto-parietal network and for sustained goal-oriented cognitive control via the cingulo-opercular network (Dosenbach et al., 2007; Draganski et al., 2008).

Brain regions associated with the “fronto-parietal” network include the dorsolateral prefrontal cortex (dlPFC), the inferior parietal cortex (IPC), the dorsal precuneus (DPC), precuneus and middle cingulate cortex (mCC; Dosenbach et al., 2007). The dlPFC is a key region in the “fronto-parietal” network and is implicated in the integration, maintenance and manipulation of goal relevant information in working memory (Fuster and Alexander, 1971; Barbey et al., 2013). Anterior aspects of the dlPFC are involved in attentional switching, selective attention and sustained attention (D’Esposito and Postle, 1999; MacDonald et al., 2000). As such, the dlPFC has been found to be associated with self-regulatory capacities such as planning, selection and initiation of action and the ability to flexibly adjust mental set (Alvarez and Emory, 2006; Clark et al., 2008). In the context of health behaviors, evidence suggests that activity in the dlPFC is associated with “on-line” processing of information linked to health behavior choices (Hare et al., 2011), and with successful self-regulatory control (Harris et al., 2013). The initiation and flexible adjustments in control associated with the “fronto-parietal” network is complemented by activity in the cingulo-opercular network hypothesized to be involved in sustained task maintenance during cognitive control (Dosenbach et al., 2007).

Sustained goal oriented control is implemented by the set of interconnected brain regions that make up the cingulo-opercular network including the anterior prefrontal cortex (aPFC), the anterior ventrolateral prefrontal cortex (aVPC), and the dorsal anterior cingulate/medial superior frontal cortex (dACC/msFC). The aPFC is a key region in the cingulo-opercular network and is involved in the integration of working memory with the allocation of attentional resources in the pursuit of higher order behavioral goals (Ramnani and Owen, 2004). Activity in the aPFC is associated with the ability to keep in mind over time a high level goal while performing associated subgoals (Koechlin and Hyafil, 2007), an important regulatory process in the pursuit of physical activity goals. It is therefore likely that activity in the aPFC will be associated with physical activity and sedentary behavior.

Another key region in the cingulo-opercular network is the ventrolateral prefrontal cortex (VLPFC). Activity in the VLPFC has been implicated in cognitive control of memory (Badre and Wagner, 2007) and in response selection and inhibition (Aron et al., 2004; Badre and Wagner, 2004). The left anterior VLPFC has been associated with the retrieval of relevant knowledge from memory (Badre and Wagner, 2007). The right VLPFC plays a central role in exerting both intentional and incidental self-control across multiple domains, particularly in conflict situations between goal-directed intentions and a prepotent impulse (Cohen et al., 2013). Activity in the right VLPFC is associated with the ability to inhibit habitual responses to cues or to inhibit a previously rewarded response in order to be able to make a different one (Cohen et al., 2013). Greater activity in the right VLPFC is associated with choosing delayed goal-related options (Monterosso et al., 2007). Regularly active individuals—those that complete at least 30 min of activity five times per week, or 20 min of vigorous activity three times per week—are likely characterized by exercise decisions that preferentially favor distal benefits (e.g., health benefits). Sedentary individuals may be distinguished by work and/ or leisure priorities that involve prolonged sitting or by the tendency to indulge immediately available passive rewards (e.g., relaxation, comfort). To some degree, it is likely that engaging in regular physical activity is associated with activity in the VLPFC. Moreover, given that the right VLPFC is activated in situations where people have to inhibit a previously rewarded response in order to be able to make a different one, it is possible that endeavors to change sedentary behavior may also be associated with activity in the right VLPFC.

In sum, the cingulo-opercular and the fronto-parietal networks work together to support executive functions that enable flexible and stable cognitive control of goal-directed behavior (Dosenbach et al., 2007). Additionally, engaging in goal pursuit activates several representational processes simultaneously, some of which might conflict, and only one of which can be expressed behaviorally at any time (Berkman and Lieberman, 2009). Thus, a control mechanism is required for signaling discrepancy between a current state and desired end-state, and in situations when processes produce conflicting responses.

## Cognitive control of goal conflicts

The anterior cingulate cortex (ACC) is a primary cortical area implicated in situations of conflict among multiple representations of response options or among actual response behaviors (Carter et al., 2000; Botvinick et al., 2001; Paus, 2001). The ACC is interconnected with cortical and subcortical (limbic-amygdala) brain regions and is active in the modulation of cognitive (dACC) and emotional (vACC) processing (Bush et al., 2000). The ACC evaluates and monitors for conflict and errors that signal the need to adjust control, particularly when a response requires selection among conflicting alternatives or when incongruity exists between intended and actual response (Bush et al., 2000; Botvinick et al., 2001, 2004; Holroyd and Coles, 2002). The ACC has dense connections with lateral frontal and parietal structures such that, in situations of conflict the ACC recruits the dlPFC to resolve conflicts and guide behavior (Kerns et al., 2004). The links between the ACC and the thalamus and brain stem nuclei implicate the ACC in arousal and drive states, and projections from the ACC to the motor cortex and the spinal cord implicate the ACC in aspects of motor control (Paus, 2001). The functional overlap of ACC connected domains implicates the ACC in both the initiation of action and in overriding competing alternatives (Paus, 2001). Thus, executive functions required to translate intention into action are supported by the ACC.

In the context of physical activity, Hall et al. (2008) used a Stroop task, a measure of response inhibition, wherein incongruent color–word pairs only were presented, and functional imaging to examine the role of ACC in individuals successful and unsuccessful at consistently translating their physical activity intentions into action. They found that unsuccessful self-regulators, i.e., those who were characterized by low intention-behavior consistency, demonstrated a significantly higher degree of activation in the ACC when performing the incongruent Stroop task relative to successful self-regulators, i.e., individuals characterized by high intention-behavior consistency. These findings suggest that, unsuccessful self-regulators, display greater recruitment of cognitive resources in the ACC, as indicated by increased activation of error detection and deliberative processes in response to the cognitive challenge posed by the Stroop task. However, the ACC is seldom activated in isolation of other regions involved in self-regulatory control, such as the orbitofrontal cortex (OFC; Walton et al., 2007). The OFC is functionally connected to prefrontal (dlPFC, ventrolateral PFC) and subcortical (e.g., basal ganglia, amygdala) regions, and this connectivity enables the OFC to act as an interface between affective information and symbolic processing associated with prefrontal regions. The OFC appears to be involved in evaluating the motivational or emotional significance of incoming information (Krawczyk, 2002; Ramnani and Owen, 2004), and is activated when people make long-term decisions (Wallis, 2007), as well as when they attend to consequences of actions (Walton et al., 2007).

## An integrated system of self-regulation

The cognitive-control networks and the DMN operate as a functional system in the control of external and internal attention (Vincent et al., 2008; Gerlach et al., 2011). It appears that a key role of the DMN is to dynamically link self-reflection with goal-directed activities through interacting neurocognitive networks (Treserras et al., 2009; Vann et al., 2009; Voss et al., 2010). Within these networks, the right anterior insula has been shown to play a key role in switching between the DMN and cognitive-control networks (Menon and Uddin, 2010). Increased connectivity at rest between the right insula and elements of the cognitive-control network has been shown to support more efficient switching between the DMN and control networks (Hasenkamp and Barsalou, 2012; Tang et al., 2012). Efficient co-activation and/or switching between these brain states is crucial for self-regulation. Given evidence that individual differences in regulatory activity between the default mode and cognitive-control network is associated with variability in goal-directed behavior (Kelly et al., 2008), it is quite possible, that to some degree, physical activity and sedentary behavior may emerge from differences in these interconnected networks. Differences in the nature and strength of brain activation during self-regulatory tasks and differences in the level of connectivity between the DMN and the cognitive-control network may underpin self-regulatory processes that influence physical activity and sedentary behaviors.

## Cognitive control and reward processing in health decisions

People often evaluate the rewarding properties (value) of a behavior when making decisions about whether to engage in it. The ability to anticipate the reward value of physical activity and then use that information to develop and execute an action plan efficiently is likely to partially underlie physical activity behavior. This idea is supported by research suggesting that in contexts individuals consider rewarding, cognitive control can be increased (Pochon et al., 2002; Locke and Braver, 2008). In these contexts, increased activity in the dlPFC guides anticipatory implementation of behavioral goals within working memory (Jimura et al., 2010) and increased activity in the frontopolar PFC is associated with maintaining an overall goal while simultaneously performing tasks linked to related subgoals (Koechlin et al., 1999; Braver and Bongiolatti, 2002). For individuals who consider physical activity a rewarding behavior, increased recruitment of brain regions that support cognitive control may enable them to sustain attention on their long-term exercise goals, experience fewer lapses in control and exert more effort to maximize the health benefits attained from physical activity.

The health benefits of physical activity accrue over the long term, thus making decisions regarding physical activity and sedentary behavior requires a balance between reward-seeking and inhibitory control processes. Cognitive control processes are instrumental in determining whether individuals make healthy choices. Cognitive control prefrontal regions are densely interconnected with brain regions and pathways associated with reward (vmPFC, amygdala, ventral and dorsal striatum, mesocortical and mesolimbic). Differential contributions of the PFC and subcortical networks underlie self-regulation success and failure (Bruce et al., 2011; Heatherton and Wagner, 2011). Indeed, effective connectivity between prefrontal and subcortical regions associated with intertemporal choice has been shown to predict individual differences in the self-control of health behaviors (Hare et al., 2014). Successful self-regulation occurs to the extent that the prefrontal-subcortical balance favors control-related PFC regions that support goal-directed action over competing subcortical activity linked to impetuous behaviors. Shifting the balance from activity in prefrontal regions to subcortical limbic structures, either due to habitual cue reactivity, or impaired prefrontal function (e.g., relatively lower cognitive control ability, negative mood) precipitates self-regulation failure (Heatherton and Wagner, 2011).

Cognitive regulation occurs through two mechanisms; value modulation in which the value (reward) assigned to a stimulus is changed and behavioral control in which the weight given to the assigned value during the action selection process is changed (Hutcherson et al., 2012). Neuroimaging research suggests that the vmPFC, the dlPFC and the inferior frontal gyrus (IFG) operate as a valuation circuit (Aron et al., 2004; Hare et al., 2009; Jasinska et al., 2011). The vmPFC is involved in computing the subjective value of a goal and in using this value to bias subsequent behavioral choice (Rangel and Hare, 2010; Jasinska et al., 2011). The dlPFC is involved in modulating the vmPFC so that its activity takes into account the value of long-term, abstract goals (e.g., be healthy; Hare et al., 2014). The IFG is implicated in interference resolution and response inhibition (Aron et al., 2004).

Recent findings from a series of studies on healthy food choices by Hare et al. (2011); Harris et al. (2013) and Hare et al. (2014), showed that modulation of the vmPFC by the dlPFC is critical in making decisions that are consistent with long-term health goals. For example, Hare et al. showed that immediately rewarding aspects of food (e.g., tastiness) are preferentially incorporated into values computed by the vmPFC, whereas more abstract values (e.g., healthiness) are only represented strongly if the dlPFC comes online and modulates activity in vmPFC so that it weights all attributes according to high level goals (e.g., make healthier choices). Additionally, they found that although the vmPFC encoded the value of food attributes at the time of choice for both successful and unsuccessful self-regulators, a pattern of increased effective connectivity between the dlPFC and the vmPFC was exhibited only for individuals successful at exerting dietary self-control. In successful self-regulators, increased activity in the dlPFC likely reflects the engagement of executive functions such as, inhibiting or attenuating hedonic stimulus attributes and allocating more attention to long-term health goals, thus enabling these individuals to choose delayed rewards. The extent to which people are able to exert cognitive control to prioritize the long term value of physical activity over immediate rewards is likely associated with physical activity behavior. It is possible that differences in the efficiency of functional connectivity of the dlPFC–IFG–vmPFC network may be associated with physical activity levels.

A key source of self-regulation failure may be the tendency of the valuation circuit to disproportionately weigh immediate rewards as more valuable comparative to delayed future rewards (McClure et al., 2004; Mitchell et al., 2011). Without sufficient activity in prefrontal cognitive control regions to modulate activity in reward circuits the balance of neural activity may shift from prefrontal to subcortical reward systems, such as the mesolimbic dopamine system- associated with choosing immediate rewards and impulsive behavior (Heatherton and Wagner, 2011). Subcortical reward systems can carry opposing sources of information from those originating in the PFC (Miller and Cohen, 2001; Heatherton and Wagner, 2011). Sedentary behavior may be partially a reflection of self-regulation failure resulting from an increased sensitivity to the rewarding effects associated with sitting and inactivity-related cues (e.g., TV, computer, and elevator) and compromised cognitive control ability to exert strategic attention or inhibit impulses in the presence of such cues.

In sum, the aforementioned research suggests that effective modulation of activity in subcortical reward regions by prefrontal networks that support cognitive control is central to making decisions that are consistent with long term health goals. In line with accumulating evidence of training induced cognitive and neural plasticity (Karbach and Schubert, 2013), training executive control abilities may enhance cognitive control processes to better modulate and bias the value of physical activity in line with long-term benefits.

## Neuroplasticity and training for transfer

The reciprocal relationship between cognitive control and physical activity implies that exercise and/or cognitive training may elicit mutually beneficial outcomes. Both aerobic and resistance training programs (Kramer et al., 1999a; Colcombe and Kramer, 2003; Liu-Ambrose et al., 2010a,b; Erickson et al., 2011) have been shown to be effective at improving cognitive control across the lifespan. This work suggests that significant training-induced cognitive plasticity can occur during aging. Combined physical activity and cognitive training interventions, either sequentially or simultaneously, has also been shown to lead to improvements in cognitive control in adults with and without cognitive impairment (Lawla et al., 2014).

Recent research has demonstrated the antecedent role of cognitive control in the self-regulation of physical activity (Best et al., 2014). This work builds on prior evidence linking greater cognitive control to subsequent higher levels of physical activity (Hall et al., 2008; McAuley et al., 2011). Specifically, Best et al. (2014) found that older women who were involved in exercise training, and who also improved their executive function, subsequently maintained higher levels of physical activity over a following 12-month period.

Increasingly, research shows that cognitive control abilities are malleable, and that cognitive training can produce positive cognitive outcomes and improvements in daily function (Willis et al., 2006; Hertzog et al., 2008) that can have long-lasting effects (Rebok et al., 2014). Approaches to cognitive training are numerous and varied; however, the relative superiority of different approaches with regard to training and transfer continue to be debated. One avenue that is garnering empirical evidence for augmenting cognitive control of physical activity is computerized training of cognitive control abilities. Research suggests that computerized training provides an effective and less labor-intensive approach for enhancing executive functions (Kramer et al., 1999b; Kueider et al., 2012). Findings from computerized cognitive training interventions show that, among older healthy older adults, mind-body aerobic exergaming (Anderson-Hanley et al., 2012), dance-step video games (Schoene et al., 2013), exergaming with a dual-task component (e.g., Microsoft Kinect-controlled Sudoku; Kayama et al., 2014), and stationary computer-based dual-task paradigms lead to modest improvements in cognitive functioning as well as positive transfer effects on balance and gait speed (Li et al., 2010; Verghese et al., 2010).

Recent research suggests that cognitive training efforts may be more effective if they contain key components such as feedback, adaptive progression, multitasking and training sessions spaced over time, that are specifically tailored to the domain of interest (Anguera et al., 2013; Mishra and Gazzaley, 2014; Wang et al., 2014). Moreover, evidence suggests that uni-modal interventions (i.e., focusing on one aspect of functioning) are more effective at generating improvements in individual cognitive abilities whereas multi-modal interventions may show greater transfer effects (Cheng et al., 2012). Understanding the components of cognitive training programs with the most potential to generate positive cognitive outcomes and improve transfer effects to real-world contexts will have important implications for designing interventions to promote physical activity regulation.

For cognitive training to be effective, it is important that training not only benefits the specific cognitive control abilities that are trained but that training can be transferred to other similar abilities (near transfer effects) as well as those more distally associated with the trained cognitive abilities (far transfer effects; (Barnett and Ceci, 2002). Evidence of the extent to which cognitive training improvements show positive transfer on real-world outcomes is variable (Hofmann et al., 2012; Wang et al., 2014). In general, transfer effects of cognitive training, and in particular far transfer, can be difficult to demonstrate. A majority of studies documenting transfer effects report positive transfer to tasks similar to the trained task (i.e., near transfer; Dahlin et al., 2009). Anguera et al. (2013) found that training on a multitasking video game customized for older adults led to positive changes in cognitive control abilities, with improvements comparable to those observed in younger adults who are habitual video-game players (see also Kramer et al., 1999c). This work suggests that customizing cognitive training programs to specific populations may increase the likelihood of observing transfer effects. There is some evidence of positive far transfer effects of cognitive training. For instance, Jaeggi et al. (2008) found that training on a working memory task (*n*back) transferred to improvements in fluid intelligence, and more time spent in training led to greater transfer effects on fluid intelligence (but see Harrison et al., 2013).

Emerging research suggests that training cognitive control abilities may translate to improved self-regulation, particularly for individuals with low executive function (see Hofmann et al., 2012). To date, no research has examined whether cognitive training may have transfer effects on the self-regulation of physical activity behavior. Nonetheless, evidence that increased executive ability for inhibition predicted short-term exercise participation in young adults (Hall et al., 2008), and evidence that multi-tasking and inhibition abilities were significant predictors of subsequent long-term exercise adherence through the mediation of self-efficacy (McAuley et al., 2011) suggests that training cognitive control abilities holds significant potential for improving self-regulation for physical activity. In a similar vein, findings from an exercise intervention that combined visio-spatial training with thought-suppression demonstrated a simultaneous increase in adherence to an exercise program designed with periodization, i.e., more frequent sessions over time (Oaten and Cheng, 2006).

Interventions designed to increase cognitive control with transfer to physical activity-specific regulation may be more effective if they included components such as, stationary or motion-based computerized training tasks that activate mind-body connections, or tasks that concurrently enhance cognitive processes and motor movements fundamental to autonomous physical activity training. Interventions that include dual activation of movement and reward systems may also be effective at increasing training transfer to physical activity-specific regulation. Further, the utility of cognitive training for physical activity regulation may be enhanced by tailoring programs to specific executive function deficits for particular exercise subpopulations. For instance, for sedentary individuals, training efforts could be focused on cognitive abilities (e.g., inhibition, strategic attention, task-switching) that transfer to improved self-regulatory skill for initiating and maintaining exercise, and for overriding temptations to remain sedentary whereas in frail older adults, training efforts could focus on balance or gait dual-task training that transfer to improved lower-body functioning. In sum, interventions that augment cognitive control abilities provide exciting and important new avenues for enhancing self-regulation capacities for physical activity.

## Conclusions and recommendations for future research directions

It has become clear that multiple brain networks including the DMN, the fronto-parietal and cingulo-opercular networks involved in cognitive control, and brain regions and pathways associated with reward (vmPFC, amygdala, ventral and dorsal striatum, mesocortical and mesolimbic) are functionally interconnected in the service of self-regulation. Neural activity associated with cognitive control is orchestrated by prefrontal regions. In accord with Hall and Fong (2010, 2013) temporal self-regulation theory, it appears that differences in executive control abilities and in the efficiencies of cognitive control networks that drive them, play a primary role in how well people are able to direct their attention to goal relevant information whilst restricting or inhibiting attention to competing goals, impulses, intrusive thoughts or emotions. These differences correlate with variability in self-regulation capacity. From this perspective, self-regulation is construed as behavioral manifestations of cognitive control processes (see also Nes et al., 2009; Kaplan and Berman, 2010; Hofmann et al., 2012). It is also clear that cognitive control abilities play a consequential role in the self-regulation of physical activity and sedentary behavior. Appropriate allocation of attentional resources and flexible cognitive control may be essential for maintaining a less sedentary and more physically active lifestyle. Although exercise self-regulation success is enhanced by supportive social networks and by environments that contain cues that encourage physical activity, without a functional threshold of cognitive control efficiency, individuals’ attempts to process new exercise information, resist inactivity urges, overcome mental fatigue during exercise, and reprioritize plans to continue an exercise program are unlikely to be successful.

Training cognitive control abilities holds significant potential for improving self-regulation in sedentary individuals. Indeed, greater reductions in sedentary behavior are more likely to be realized by the addition of cognitive training components to interventions designed to decrease sedentary behaviors and increase physical activity. Given that cognitive control abilities work in concert to facilitate multiple aspects of self-regulation, optimizing cognitive control capacities will necessarily depend not only on exercising particular executive functions but training them in a coordinated fashion that involve interactions among individual capacities.

Despite recent advances, we do not fully understand the cognitive mechanisms that lead to successful exercise self-regulation and those that precipitate self-regulation failures that predispose people to remain sedentary. In particular, there is a pressing need to further identify the coordinated neural networks, which underlie the cognitive control of physical activity regulation. Increasing the utilization of individual differences approaches in future studies would advance our understanding of what aspects of cognitive control and neural systems are compromised in sedentary individuals. Greater knowledge of cognitive control deficits that underlie sedentary behavior would improve future intervention efforts to increase exercise self-regulation in regularly sedentary individuals. Identifying structural changes in the brain and the neural mechanisms of neuroplasticity by which cortical representations are functionally remodeled as a consequence of cognitive training, and, how these changes correlate with observable self-regulatory behavior is another potential avenue for future research. Further, future studies using longitudinal designs are required to gain insight on causal relations between cognitive control and self-regulation success and failure for physical activity.

The present review presented emerging research demonstrating the instrumental role of cognitive control abilities in self-regulation success and failure for physical activity. Additionally, neural networks that may underpin the cognitive control of physical activity and sedentary behavior were proposed. Cognitive training interventions for physical activity and key components of training program that may yield positive cognitive outcomes associated with increased physical activity were reviewed. The integration of exercise neuroscience and behavioral medicine fields holds significant potential to generate new knowledge that can be used to enhance cognitive control abilities that increase self-regulation capacity for physical activity.

## Conflict of interest statement

The authors declare that the research was conducted in the absence of any commercial or financial relationships that could be construed as a potential conflict of interest.
